# Simultaneous right-sided nephrectomy with orthotopic liver and kidney transplantation—An alternative method for patients with autosomal dominant polycystic liver and kidney disease

**DOI:** 10.1007/s00423-021-02206-9

**Published:** 2021-05-26

**Authors:** Philipp Felgendreff, Hans-Michael Tautenhahn, Sascha Lux, Felix Dondorf, René Aschenbach, Falk Rauchfuss, Utz Settmacher

**Affiliations:** 1 Department of General, Visceral and Vascular Surgery, University Hospital Jena, Am Klinikum 1, 07747 Jena, Germany; 2 Research Programme “Else Kröner-Forschungskolleg AntiAge”, University Hospital Jena, Am Klinikum 1, 07747 Jena, Germany; 3 Department of Radiology, University Hospital Jena, Am Klinikum 1, 07747 Jena, Germany

**Keywords:** Kidney transplantation, Liver transplantation, Autosomal dominant polycystic liver and kidney disease

## Abstract

**Purpose:**

In patients suffering from autosomal dominant polycystic liver and kidney disease (ADPLKD), combined organ transplantation often poses a technical challenge due to the large volume of both organs. To simplify the transplantation procedure by improving the exposure of anatomical structures, we introduce a novel surgical technique of orthotopic liver and kidney transplantation.

**Methods:**

The modified simultaneous liver and kidney transplantation technique via a right-sided L-incision included three steps: (1) right-sided nephrectomy in the recipient followed by (2) orthotopic liver transplantation in cava replacement technique and (3) the orthotopic kidney transplantation with arterial reconstruction to the right common iliac artery.

**Results:**

In total, seven patients with ADPLKD were transplanted by using the modified transplantation technique. The mean operation time was 342.43 min (±68.77). Postoperative patients were treated for 6.28 days (±2.50) in the intensive care unit and were discharged from the surgical ward approximately 28 days (±5.66) after the operation with normal graft function. Complications associated with the use of the modified technique, such as bleeding, anastomotic stenosis, biloma, or urinoma, did not occur.

**Conclusion:**

Modified simultaneous liver and kidney transplantation is a safe alternative for patients with ADPLKD. By combining right-sided nephrectomy and orthotopic graft transplantation, the approach optimizes the exposure of anatomical structures and simplifies the transplantation procedure. Additionally, the modified transplantation technique does not require a particular organ explantation procedure and can be applied for all liver and kidney grafts.

## Introduction

Since first reported by Margreiter et al*.* in 1984 [1], the simultaneous transplantation of the liver and kidney has been accepted as an effective surgical method in clinical practice, with good patient outcomes [2] and significant improvements in quality of life [3].

One indication for simultaneous organ transplantation is autosomal dominant polycystic liver and kidney disease (ADPLKD) (Potter III). In the presence of ADPLKD, massive enlargement of the liver and kidney caused by the cysts [4] can occur in up to 78% of patients [5, 6]. These patients mainly suffer from nonspecific clinical symptoms due to the massive organ enlargement, the position and size of individual cysts. The therapeutic procedure for ADPLKD is usually aligned with Gigot’s classification [7], aiming primarily at symptom control as well as maintaining organ function. In 42% of all patients with type II and III ADPLKD, simultaneous liver and kidney transplantation are necessary [8, 10].

Generally, the clinical standard transplantation procedure is based on two steps: (1) orthotopic liver transplantation and (2) heterotopic kidney transplantation in the iliac fossa via a second surgical incision (two-step cLKTx). The massive enlargement of the affected organs in patients with ADPLKD makes this combined transplantation process complex. Thus, the technique and timing of simultaneous liver and kidney transplantation for ADPLKD are subject to individual variability [11] and the center-specific approach [12–14].

The key issue for all existing surgical techniques is to reduce the clinical symptoms resulting from increased polycystic organ volume and to simplify the transplantation procedure by improving the representation, scope, and details of the anatomical structure.

In this article, we present a new modified surgical technique for orthotopic simultaneous liver and kidney transplantation to address this issue in patients with ADPLKD. In addition to the exact surgical protocol and the postoperative follow-up, the safe feasibility of this modified technique is also documented for each patient who underwent transplantation in our center since September 2016.

## Methods

### Preoperative clinical parameters

The modified surgical technique of simultaneous liver and kidney transplantation has been used at the Transplantation Center of the Jena University Hospital, Germany, since September 2016. The indication for the modified simultaneous liver and kidney transplantation technique was patients with ADPLKD showing at least a doubled physiological liver [15] and kidney [16] volume according to preoperative diagnostics as well as ADPLKD-associated complications.

The respective patients underwent a comprehensive evaluation for possible contraindications. Furthermore, the internal interdisciplinary transplantation committee approved the indication for simultaneous organ transplantation based on the current regulations [9] prior to listing.

The preoperative clinical parameters of the patients were derived from the internal hospital data reporting system (SAP (SAPGUI 740, SAP SE® Walldorf, Germany), Copra 5 (Version v5.24.974, COPRA System GmbH, Sasbachwalden, Germany), and Copra 6 (Version v6.7.20, COPRA System GmbH, Sasbachwalden, Germany) as well as from the data transmitted to EUROTRANSPLANT. The characteristics of the donors and the recipients, along with the results of routine clinical examinations, were evaluated in the reported data analysis (ethical committee application number: 4428-05/15).

### The modified simultaneous liver and kidney transplantation technique

After the induction of general anesthesia according to the global guidelines [17, 18], the abdominal cavity of the recipient was opened with a right-sided L-incision.

The L-incision included a vertical and a right horizontal abdominal laparotomy. The vertical laparotomy runs from the xiphoid down towards the umbilicus. Four fingers above the umbilicus, the vertical laparotomy was turned in a right horizontal direction to the center between the costal arch and the anterior superior iliac spine.

The abdominal cavity was exposed via bilateral retraction of the ribcage to obtain access to the liver and the right kidney. During exploration, selected cysts were opened to allow the mobilization of the organs and preparation of the anatomical structures.

Right-sided nephrectomy of the polycystic altered organ was performed first to facilitate hepatectomy, to simplify the transplantation procedure, and to reduce the intra-abdominal volume. In preparation for this step, the right-sided colon and the duodenum were mobilized, and the renal vein and artery were identified and dissected. During nephrectomy, special attention must be paid to maintain a long ureter with a surrounding mass of vessel-supplied tissue.

Next, the liver was mobilized and prepared for hepatectomy, including the retrohepatic vena cava. Before hepatectomy, the liver and kidney grafts were prepared on the back table for implantation.

Subsequently, the liver transplantation procedure was continued, following its usual course with the caval replacement technique and without the use of a temporary portocaval shunt or veno-venous bypass. Reperfusion, both portal and arterial, was performed simultaneously. The bile duct was reconstructed with duct-to-duct modified end-to-end anastomosis.

After the completion of liver transplantation, kidney transplantation in the orthotopic retroperitoneal position was performed via the pre-established right-sided L-incision. The venous anastomosis was made directly to the inferior vena cava below the original stump of the right renal vein. Arterial reconstruction was established in the right common iliac artery of the recipient using an arterial interposition graft of the donor iliac artery, which was guided retroperitoneally. After reperfusion of the kidney graft and preparation of the recipient ureter, an end-to-end uretero-ureterostomy was performed with double-J catheter splinting. The established L-incision was closed after inserting drainage tubes (two 20-gauge French tubes were placed subhepatically and subphrenically).

### CT scan and ultrasound imaging

**Fig. 1:**
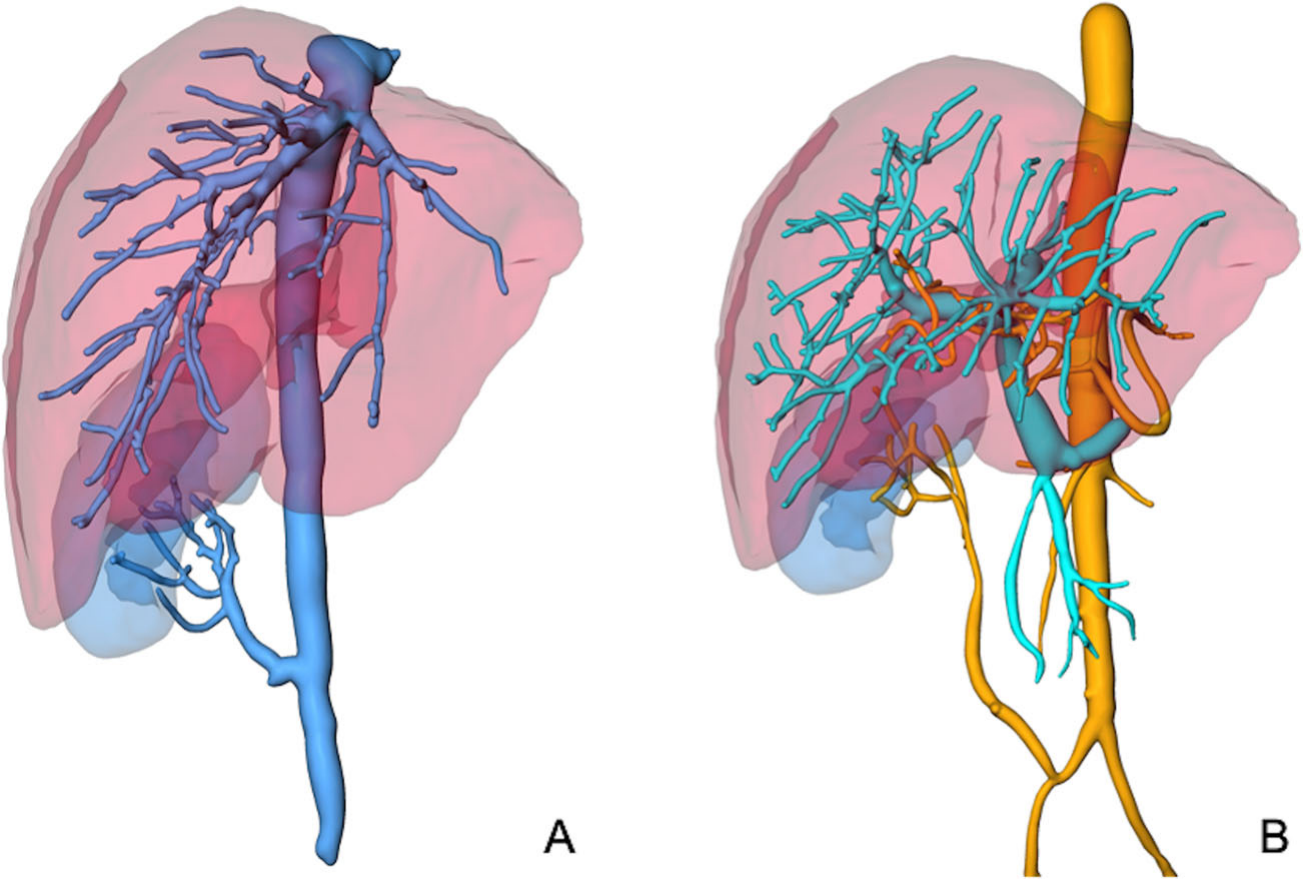
CT reconstruction (MeVis® (Frauenhofer MeVis, Lübeck, Germany)) after transplantation in a patient with polycystic liver and kidney degeneration. **A**: Illustration of the venous vascular anatomy of the transplanted organs. **B**: Illustration of the arterial and portal venous anatomy of the transplanted organs

As part of the routine evaluation procedure, full-body computed tomography (CT scan; CT Revolution 256 slice, GE Healthcare, USA) was performed for each potential recipient. The organ volumes of the polycystic liver and kidney were calculated using Cerner SkyVue® Distribution (Version 2014.01.05, Cerner, USA) and the available CT scan results. To visualize the transplanted organs and their vascular supply, clinically indicated postoperative CT scans were reconstructed with MeVis® (Frauenhofer MeVis, Lübeck, Germany) (Fig. 1). To evaluate the postoperative course, the recipients underwent routine ultrasound examinations (Vivid S70, GE Healthcare, USA). During these examinations, perfusion of the transplanted organs was examined by determining the arterial and venous blood velocity and arterial vascular resistance.

## Results

### Preoperative clinical parameters

Since 2016, the modified simultaneous liver and kidney transplantation technique has been the standard operation technique for patients suffering from ADPLKD at the Transplantation Center of Jena University Hospital. Until October 2018, a total of seven patients were treated using this modified technique.

**Fig. 2:**
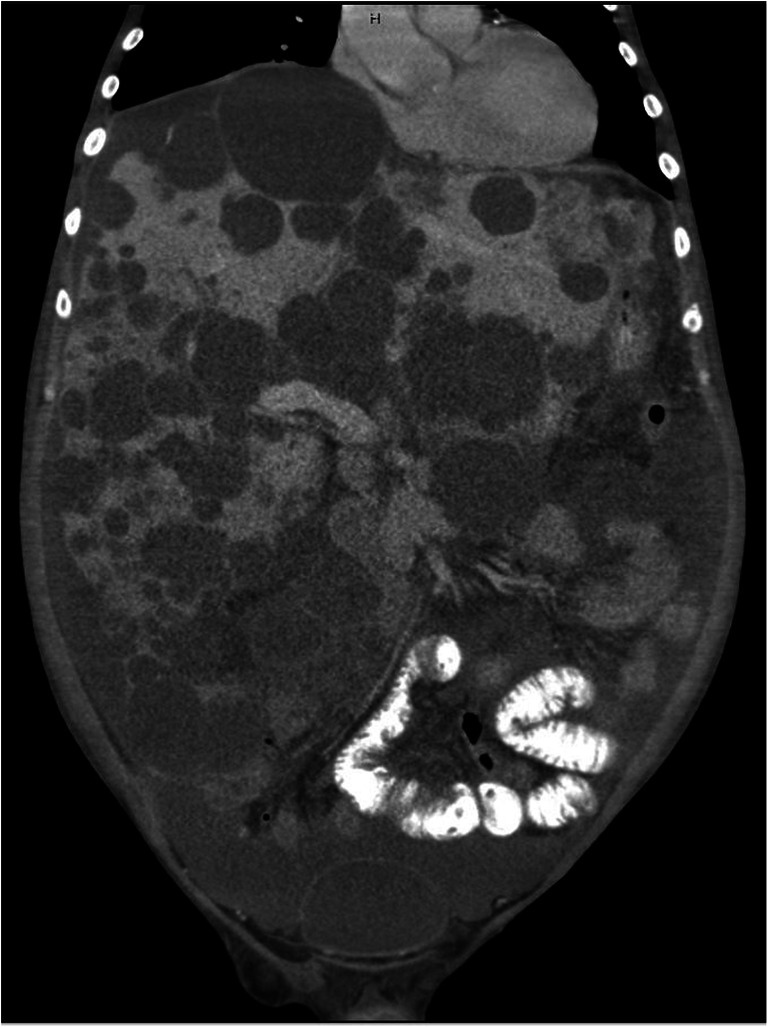
Coronal presentation of the portal vein in a polycystic liver. The kidneys are also polycystically degenerated as seen in the CT scan (CT Revolution 256 slice, GE Healthcare, USA) before transplantation

**Fig. 3:**
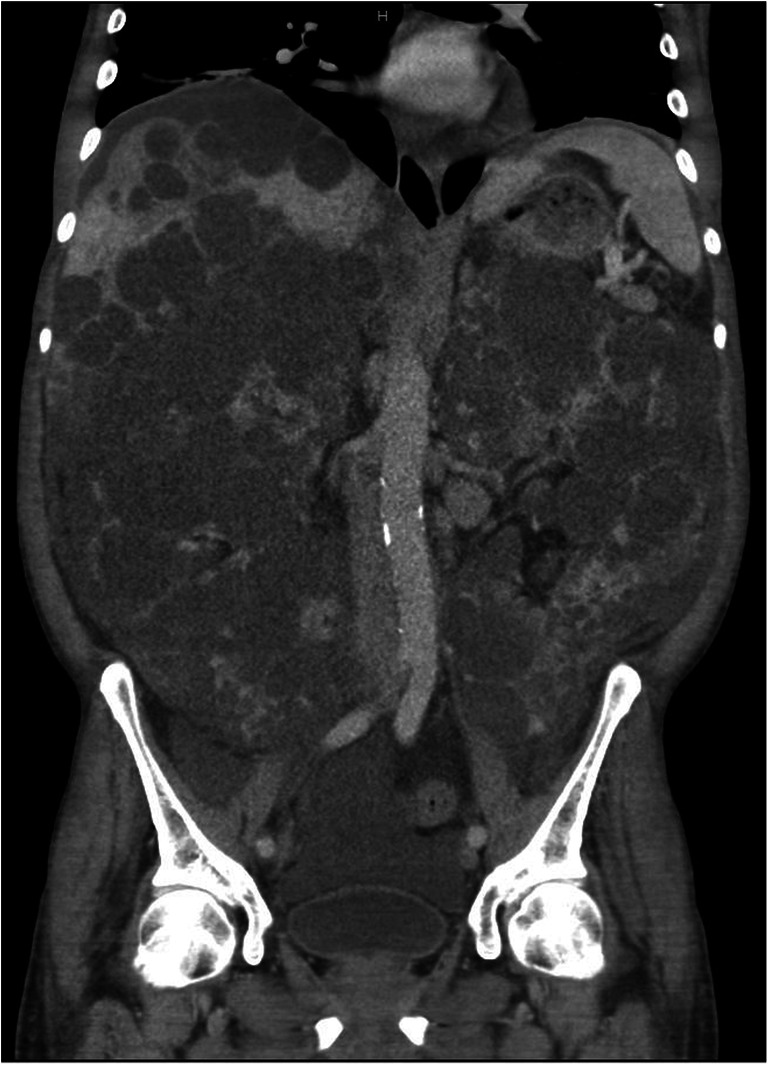
Coronal presentation of the caval region showing polycystic alterations in both kidneys on the CT scan (CT Revolution 256 slice, GE Healthcare, USA) before transplantation

At the time of transplantation (Table 1), the patients were on average 54.14 years (±3.98) old. Four of them were female. All patients had restricted liver function with a laboratory Model of End-Stage Liver Disease score (lab-MELD) of 18.57 points (±2.37). All patients fulfilled the standard exceptional criteria for prioritization on the waiting list according to the EUROTRANSPLANT manual (Standard Exceptional Model of End-Stage Liver Disease (SE-MELD): 29.29 points (±2.06)) [19]. The average waiting time prior to transplantation was 834.57 days (±832.23). The patients were categorized as ASA III with comorbidities such as normochromic normocytic anemia, renal hypertension, and type 2 diabetes. The mean body mass index (BMI) of the patients was 27.09 kg/m^2^ (±5.59). During the routine evaluation procedure, morphological analysis through a CT scan revealed an average liver volume of 9343 ml (±3698.56) and an average right kidney volume of 2770 ml (±1128.78) (Table 2). All patients were categorized as type III according to Gigot’s ADPLKD classification (Figs. 2 and 3). The donor-specific parameters including sex, age, BMI, and extended donor criteria are presented in Table 2.

**Table 1:** Preoperative clinical parameters of patients with ADPLKD evaluated for modified liver and kidney transplantation

	Patient-ID							Average (SD)
	P-001	P-002	P-003	P-004	P-005	P-006	P-007	
Age (y)	52	50	57	54	61	55	50	54.14 (±3.98)
Waiting time (d)	781	589	1032	2602	252	250	336	834.57 (±832.23)
Sex	Male	Female	Female	Male	Female	Female	Male	-
Preoperative dialysis	No	No	Yes	No	No	No	No	-
Body weight (kg)	77	53	73	117	73	83	84	80 (±19.28)
Body height (m)	1.83	1.56	1.68	1.79	1.62	1.60	1.95	1.72 (±0.14)
BMI (kg/m^2^)	23.10	21.80	25.90	36.50	27.82	32.42	22.09	27.09 (±5.59)
Blood type (*)	0 (−)	0 (+)	0 (+)	A (+)	0 (+)	0 (+)	0 (+)	-
Lab-MELD	17	15	20	19	20	17	22	18.57 (±2.37)
SE-MELD	29	33	29	29	26	29	30	29.29 (±2.06)

Values are presented as the average and standard deviation (SD); *Rhesus-factor; *BMI*, body mass index; *Lab-MELD*, Laboratory Model of End-Stage Liver Disease; *SE-MELD*, Standard Exceptional Model of End-Stage Liver Disease

**Table 2:** Donor-, intraoperative-, and organ-specific parameters in patients with modified liver and kidney transplantation

		Patient-ID							Average (SD)
		P-001	P-002	P-003	P-004	P-005	P-006	P-007	
Donor-specific parameter	Age	55	79	61	72	63	61	51	-
	Sex (M/F)	M	F	F	F	F	F	F	-
	Body weight (kg)	90	60	63	80	70	72	60	-
	Body height (m)	1.90	1.60	1.70	1.65	1.70	1.67	1.60	-
	BMI (kg/m^2^)	24.90	23.40	21.80	29.40	24.20	25.80	23.40	-
	Extended donor	Yes ^(1)^	Yes ^(2, 3)^	Yes ^(1, 3)^	Yes ^(1, 2)^	Yes ^(3–5)^	Yes ^(3)^	Yes ^(5)^	-
Intraoperative parameter	Operation time (min)	326	236	288	413	322	419	393	342.43 (±68.77)
	CIT liver (min)	420	516	535	497	395	621	208	456 (±132.46)
	WIT liver (min)	25	31	32	29	32	56	46	35.86 (±11.01)
	Cell-saver (ml)	0	400	0	335	300	250	241	218 (±158.19)
	CIT kidney (min)	559	563	648	457	488	741	550	572.29 (±95.05)
	WIT kidney (min)	12	10	21	10	20	21	34	18.29 (±8.58)
	PRC (n)	3	1	0	0	4	2	4	2 (±1.73)
	PLC (n)	0	0	0	0	0	0	0	0
	FFP (n)	0	0	0	2	3	0	0	0.71 (±1.25)
Organ-specific parameter	Liver vol. (ml)	11300	7394	6263	9867	10243	4576	15758	9343 (±3698.56)
	Kidney vol. rig. (ml)	2931	1899	1408	4336	3881	1714	3221	2770 (±1128.78)
	Liver weight (g)	4500	3600	4220	4960	5585	1775	8210	4692.86 (±1966.66)
	Kidney weight (g)	2000	1028	1900	3860	2908	984	1635	2045 (±1032)

Values are presented as the average and standard deviation (SD); CT-volumetry using CT Revolution 256 slice, GE Healthcare, USA; Cerner SkyVue® Distribution, version 2014.01.05, Cerner, USA; *M*, male, *F*, female; *BMI*, body mass index; extended donor criteria reason, ^1.^: nontraumatic subarachnoid hemorrhage, ^2.^: donor age, ^3.^: ICU stay more than 7 days, ^4.^: cancer in medical history, ^5.^: limited graft function; *WIT*, warm ischemic time; *CIT*, cold ischemic time; *PRC*, packed red cells; *PLC*, platelets; *FFP*, fresh-frozen plasma; *vol*, volume

### Intraoperative evaluation of simultaneous liver and kidney transplantation

In all seven patients, modified combined liver and kidney transplantation was performed via a right-sided L-incision. The mean operation time of the procedure was 342.43 min (±68.77) (Table 2). The establishment of the vascular liver (WIT liver) and kidney (WIT kidney) anastomoses took 35.86 min (±11.01) and 18.29 min (±8.58), respectively. On average, 785.71 ml (±393.40) of human albumin was infused to compensate for the intravascular volume. In this way, all patients were hemodynamically stable during the whole surgical procedure, including nephrectomy and hepatectomy, avoiding the use of a temporary portocaval shunt or veno-venous bypass.

In only two patients, it was necessary to compensate for the coagulopathy ROTEM driven by the transfusion of fresh-frozen plasma (FFP).

### Postoperative evaluation of simultaneous liver and kidney transplantation

After leaving the operation theater, the patients were observed in the ICU for approximately 6.28 days (±2.50). In the first seven postoperative days, unfractionated heparin (partial thromboplastin time (PTT): 40–50 s) was applicated under close surveillance of the coagulation parameters. This systemic anticoagulation was initiated to prevent thrombotic occlusions of the vascular anastomosis.

Immediate extubation and hemodynamic stabilization were achieved in all patients.

Only one case of delayed kidney function occurred. The initially impaired renal function in this patient (P-002) resulted in hypervolemia with subsequent respiratory insufficiency. After reintubation and one-off hemodialysis (Table 3; Clavien-Dindo classification IVa), kidney graft function was promptly stabilized, and the patient was transferred to the surgical ward on POD 11. During hospitalization, more than half of the patients had a minor complication, which was classified as type II according to the Clavien-Dindo classification.

**Table 3:** Preoperative, postoperative, and 3-month follow-up parameters in patients with modified liver and kidney transplantation

		Patient-ID						
		P-001	P-002	P-003	P-004	P-005	P-006	P-007
Preoperative	CREA (μmol/l)	277	257	531	356	334	360	542
	eGFR (ml/min)	21.17	18.1	7.1	15.8	12.2	11.7	9.8
	UREA (μmol/l)	17.1	15.9	14.7	4.7	20.6	18	21.8
	Hemoglobin(mmol/l)	5.7	5.9	7.5	7.6	6.5	6.3	6.5
	INR	1.1	1.0	1.0	2.1	1.1	1.1	1.1
Postoperative follow-up	Postoperative dialysis (n)	no	yes (1)	no	no	no	no	no
	PRC (n)	1	6	2	0	1	0	6
	FFP (n)	0	0	0	0	0	0	0
	Albumin (ml)	0	300	0	1000	500	0	650
	Hemoglobin (mmol/l)	5.3	4.8	5.6	4.9	5.8	6.0	5.6
	INR	1.5	1.7	1.7	1.7	1.4	2.0	1.7
	Urine secretion POD 1 (ml)	7010	3140	5160	5600	780	2800	1500
	Urine secretion POD 2 (ml)	3850	1800	2620	4100	6920	4200	3140
	Urine secretion POD 3 (ml)	1430	990	2390	2900	1440	3850	2010
	ICU stay (d)	4	11	8	4	5	6	6
	Hospitalization (d)	27	28	37	33	21	22	28
	Clavien-Dindo	II	IVa	IIIa	II	II	IIIa	II
3-month follow-up	AST (μmol/l)	0.33	0.87	0.58	0.37	0.26	0.32	0.18
	ALT (μmol/l)	0.43	1.12	0.87	0.36	0.19	0.33	0.10
	Ap (μmol/l)	1.29	2.21	1.25	0.66	1.15	1.59	1.36
	GGT (μmol/l)	0.55	2.84	0.62	0.44	0.39	2.34	0.5
	Lip (μmol/l)	0.27	0.24	0.38	0.84	0.55	1.09	0.26
	CREA (μmol/l)	112	134	73	148	83	141	164
	eGFR (ml/min)	64.7	39.8	79.1	45.6	65.9	35.9	41.2
	UREA (μmol/l)	9.2	5.1	3.5	10.7	6.3	8.3	8.9
	ALB (g/l)	39	42	34	35	38	36	32
	INR	1	0.9	1	1	1	0.9	1.1
	BILI (μmol/l)	6	24	9	6	14	4	7
	ChE (μmol/l)	150	121	161	124	117	137	73

*AST*, aspartate aminotransferase; *ALT*, alanine aminotransferase; *AP*, alkaline phosphatase; *GGT*, γ-glutamyltransferase; *Lip*, lipase; *CREA*, creatinine; *eGFR*, estimated glomerular filtration rate; *UREA*, urea; *ALB*, albumin; *INR*, international normalized ratio; *BILI*, bilirubin; *ChE*, cholinesterase; *PRC*, packed red cells; *FFP*, fresh-frozen plasma; *POD*, postoperative day

Graft function in the other six patients was promptly normalized, and no irregularities occurred during the remaining clinical course in these patients. The routine ultrasound examination showed regular postoperative results with normal perfusion parameters and no signs of cholestasis or urinary retention (Table 4). There was no relevant postoperative bleeding. The immunosuppressive rules and standards based on tacrolimus (target level 8–12 ng/ml within the first 3 months), mycophenolate, and steroids were gradually tapered and were discontinued, depending on the patient, over a period of 6 months after transplantation. Basiliximab (20 mg on POD 0 and POD 4) was used for the induction of immunosuppression.

**Table 4:** Sonographic findings using Vivid S70 (GE Healthcare, USA) in the first three postoperative days in patients with modified liver and kidney transplantation

	Parameters	Average (SD)
POD 1	PVF (cm/s)	19.14 (±8.95)
	HAF (cm/s)	59.67 (±39.02)
	RI-HA	0.55 (±0.13)
	RVF (cm/s)	25.55 (±28.35)
	RAF (cm/s)	39.17 (±13.04)
	RI-RA	0.63 (±0.07)
POD 2	PVF (cm/s)	28.33 (±10.56)
	HAF (cm/s)	44.57 (±13.64)
	RI-HA	0.58 (±0.11)
	RVF (cm/s)	12.5 (±0.71)
	RAF (cm/s)	26.33 (±8.14)
	RI-RA	0.63 (±0.06)
POD 3	PVF (cm/s)	29.52 (±16.81)
	HAF (cm/s)	41 (±19.36)
	RI-HA	0.62 (±0.08)
	RVF (cm/s)	14 (±4.24)
	RAF (cm/s)	25.42 (±6.47)
	RI-RA	0.58 (±0.05)

Values are presented as the average and standard deviation (SD); *PVF*, portal vein flow; *HAF*, hepatic artery flow; *RI-HA*, resistance index-hepatic artery; *RVF*, renal vein flow; *RAF*, renal artery flow; *RI-RA*, resistance index-renal artery; *POD*, postoperative day

Following removal of the double-J catheter, patients were discharged after 28 days (±5.66) with normal liver and kidney function (data not shown). The indication for platelet inhibition due to the transplantation procedure was not given in any of the patients. Furthermore, there was no manifestation of bile leakage, ureter stenosis, or incisional hernia during the postoperative observation period.

The postoperative pathological examination (Table 2) confirmed the preoperative calculated liver and kidney dimensions (mean liver weight 4692.86 g (±1966.66); mean kidney weight 2045 g (±1032.78). The opening of single cysts during organ explantation was responsible for the difference between the pre- and postoperative examination results.

The 3-month follow-up confirmed excellent graft function (Table 3) with normal liver synthesis and normal kidney function (retention parameters) in all patients. Indications for additional left-sided nephrectomy were not given for any patient during the follow-up period.

## Discussion

In this article, we present our first experience with a new modified surgical technique for orthotopic simultaneous liver and kidney transplantation in patients with ADPLKD.

The central steps of the modified orthotopic liver and kidney transplantation method are (1) right-sided nephrectomy of the recipient followed by (2) orthotopic liver transplantation and (3) final orthotopic kidney transplantation. Using this method, we are able to improve the transplantation procedure of patients with ADPLKD compared to the standard clinical procedure.

Our alternative surgical approach represents the further development of other innovative approaches, as recently published by Jochmans et al. [20] and Lee et al. [21].

Jochmans and colleagues perform the simultaneous organ transplantation via median laparotomy [20]. Using this protocol, the initial orthotopic liver transplantation is followed by bilateral nephrectomy. Subsequently, the kidney graft is placed in a retroperitoneal heterotopic position. For this purpose, a peritoneal pocket (15 × 15 cm) is formed along the already established surgical access (one-step cLKTx), and the graft is implanted into the iliac fossa.

Applying Jochmans’ transplantation technique reduces the mean operation time (one-step cLKTx 6.8 h (4.1–9.3 h)) by more than 2 h compared to the two-step procedure (two-step cLKTx 9.0 h (8.7–10.1 h)).

A further reduction of the mean operation time by more than 1 h can be achieved by our method (one-step cLKTx 6.8 h [20] vs orthotopic simultaneous liver and kidney transplantation 5.45 h). We assume that the additional reduction in operation time in our orthotopic liver and kidney transplantation technique is due to the initial performance of right-sided nephrectomy in the recipient. This central step just before transplantation improves the representation and detailed view of the anatomical structures during hepatectomy as well as implantation of the liver graft. Additionally, the vascular structures are openly accessible and can be prepared for subsequent kidney transplantation.

The second alternative transplantation method published by Lee et al. is indicated for patients with extensive iliac artery disease and is performed as en bloc simultaneous liver-kidney transplantation [21]. In this approach, the liver and right kidney are explanted en bloc and remain attached via the venous structures in the course of the implantation procedure. Using this protocol, the implantation process is improved by the simultaneous reperfusion of both organs. Compared to the classic technique, en bloc modification leads to a shortening of the cold (5.68 h (4.7–8.2 h)) and warm (52 min (46–57 min)) ischemic time in the kidney graft as well as a reduced total operation time (6.23 h (5.7–7.53 h)).

However, the continuous linking of the liver and kidney is accompanied by some disadvantages. The first disadvantage is arterial perfusion of the kidney graft. To reestablish arterial blood flow, extensive back-table preparation with reanastomosis of the renal and splenic arteries is essential. For this reason, Lee et al. defined a contraindication for this technique in kidney grafts with multiple renal arteries [21]. Besides, the group described a steal phenomenon and kinking of the renal artery in two patients, making major revision operations and renewed arterial anastomosis necessary.

Another disadvantage is the particular explantation procedure that enables the en bloc transplantation technique [21]. The method can only be conducted if the venous structures between the liver and right kidney transplant are maintained during the donor operation. This type of en bloc explantation is not the standard technique and can be challenging for individual surgeons. Furthermore, specific donor characteristics are required due to the missing possibility of selecting the left or right kidney in the transplantation procedure.

Besides the unique explantation technique and specific donor characteristics, further aspects need to be considered when comparing the method of Lee et al. [21] with our approach. Both methods require extensive preparation of the vascular structures. The reanastomosis of the renal and splenic arteries, as well as the use of an arterial interposition graft, is technically complex and vulnerable to complications. However, our method can also be used in case of vascular variations of the kidney graft and has no known surgical contraindication.

In summary, our modified orthotopic liver and kidney transplantation technique offers several advantages compared to the classical transplantation method as well as the approaches of Jochmans et al. [20] and Lee et al. [21]. Due to the initial right-sided nephrectomy of the recipient, the exposure of the anatomical structures is improved for hepatectomy and subsequent transplantation. In addition, the vascular structures are openly accessible and directly prepared for the final kidney transplantation, resulting in a significant reduction of mean operation time [20, 21]. Furthermore, liver and kidney transplantation can be performed through single surgical access, avoiding extensive preparation in the iliac fossa. The modified approach has no surgical contraindications and requires no specific adaptations of the explantation technique.

Nevertheless, our technique also has limitations and disadvantages. The use of arterial interposition graft requires more technical expertise and can be associated with an increased rate of complications. Moreover, depending on the orthotopic position of the kidney graft, the anastomosis of the ureter can only be executed as an uretero-ureterostomy. In reference to the European guideline for kidney transplantation, the uretero-ureterostomy is an alternative to the usual anastomosis (Lich-Gregoir or Ledbetter-Politano), though it increases the risk of complications [22].

Independently of these disadvantages, morbidity, mortality, and complication rates of our modified orthotopic transplantation technique are comparable with those of all established simultaneous transplantation techniques [20, 21, 23].

Furthermore, no surgical technique–associated complications, such as bleeding, anastomotic stenosis, biloma, or urinoma, were observed.

The interpretation of the study results is subject to specific limitations. The small size of our study is one of the major limitations. In general, the simultaneous transplantation of the liver and kidney is a very rare procedure in the EUROTRANSPLANT region. Only forty-five combined transplantations were performed in 2017 [24], but only 15% of these were indicated for ADPLKD [25].

Due to this circumstance, the evaluation of our modified procedures in a large study cohort is hampered. Compared to other studies [20, 21] and case reports [26–28], we were able to demonstrate the successful feasibility of our technique in a characteristic cohort.

Another restriction is the currently missing long-term course of our patients. There is a retrospective clinical trial planned to compare our modified orthotopic liver and kidney transplantation technique with the standard clinical heterotopic approach to evaluate our experience.

## Conclusion

In conclusion, the modified orthotopic liver and kidney transplantation technique is an alternative for combined liver and kidney transplantation. The transplantation technique simplifies the transplantation procedure without negatively influencing patient outcomes. The application of this technique for other indications of end-stage liver and kidney diseases needs to be examined but appears possible, in principle.

## Data Availability

The datasets used and analyzed during the current study are available from the corresponding author upon reasonable request.

## Abbreviations

ADPLKD: autosomal dominant polycystic liver and kidney disease
PDK-1: pyruvate dehydrogenase kinase 1
PDK-2: pyruvate dehydrogenase kinase 2
Two-step cLKTx: standard simultaneous liver and kidney transplantation
Lig: ligamentum
ICU: intensive care unit
Lab-MELD: Laboratory Model of End-Stage Liver Disease
SE-MELD: Standard Exceptional Model of End-Stage Liver Disease
BMI: body mass index
PTT: partial thromboplastin time
CT: computed tomography
CRC: concentrated red cells
FFP: fresh-frozen plasma
CIT: cold ischemic time
WIT: warm ischemic time
PVF: portal vein flow
HAF: hepatic artery flow
RI-HA: resistance index-hepatic artery
RVF: renal vein flow
RAF: renal artery flow
RI-RA: resistance index-renal artery
POD: postoperative day
AST: aspartate aminotransferase
ALT: alanine aminotransferase
AP: alkaline phosphatase
GGT: γ-glutamyltransferase
Lip: lipase
CREA: creatinine
eGFR: estimated glomerular filtration rate
UREA: urea
ALB: albumin
INR: international normalized ratio
BILI: bilirubin
ChE: cholinesterase
One-step cLKTx: modified simultaneous liver and kidney transplantation according to Jochmans et al*.*

## References

[1] Margreiter R, Kramar R, Huber C, Steiner E, Niederwieser D, Judmaier G (1984). Combined liver and kidney transplantation. Lancet (London, England) [Internet].

[2] Ueno T, Barri YM, Netto GJ, Martin A, Onaca N, Sanchez EQ, Chinnakotla S, Randall HB, Dawson S, Levy MF, Goldstein RM, Klintmalm GB (2006). Liver and kidney transplantation for polycystic liver and kidney-renal function and outcome. Transplantation..

[3] Kirchner GI, Rifai K, Cantz T, Nashan B, Terkamp C, Becker T, Strassburg C, Barg-Hock H, Wagner S, Lück R, Klempnauer J, Manns MP (2006). Outcome and quality of life in patients with polycystic liver disease after liver or combined liver-kidney transplantation. Liver Transpl [Internet].

[4] Chapman AB, Bost JE, Torres VE, Guay-Woodford L, Bae KT, Landsittel D, Li J, King BF, Martin D, Wetzel LH, Lockhart ME, Harris PC, Moxey-Mims M, Flessner M, Bennett WM, Grantham JJ (2012). Kidney volume and functional outcomes in autosomal dominant polycystic kidney disease. Clin J Am Soc Nephrol [Internet].

[5] Vauthey JN, Maddern GJ, Blumgart LH (1991). Adult polycystic disease of the liver. Br J Surg [Internet].

[6] Dumot JA, Fields MS, Meyer RA, Shay SS, Conwell DL, Brzezinski A (1994). Alcohol sclerosis for polycystic liver disease and obstructive jaundice: use of a nasobiliary catheter. Am J Gastroenterol [Internet].

[7] Gigot JF, Jadoul P, Que F, Van Beers BE, Etienne J, Horsmans Y (1997). Adult polycystic liver disease: is fenestration the most adequate operation for long-term management?. Ann Surg [Internet].

[8] Drenth JPH, Chrispijn M, Nagorney DM, Kamath PS, Torres VE (2010). Medical and surgical treatment options for polycystic liver disease1. Hepatology..

[9] Bundesärztekammer. Richtlinie gemäß § 16 Abs. 1 S. 1 Nrn. 2 u. 5 TPG für die Wartelistenführung und Organvermittlung zur Lebertransplantation. 2017;1–20.

[10] Pirenne J, Aerts R, Yoong K, Gunson B, Koshiba T, Fourneau I, Mayer D, Buckels J, Mirza D, Roskams T, Elias E, Nevens F, Fevery J, McMaster P (2001). Liver transplantation for polycystic liver disease. Liver Transpl [Internet].

[11] Moreno-Gonzalez E, Meneu-Diaz JC, Garcia I, Perez Cerdá F, Abradelo M, Jimenez C, Loinaz C, Gomez R, Gimeno A, Moreno A (2004). Simultaneous liver-kidney transplantation for adult recipients with irreversible end-stage renal disease. Arch Surg.

[12] Demirci G, Becker T, Nyibata M, Lueck R, Bektas H, Lehner F (2003). Results of combined and sequential liver-kidney transplantation. Liver Transpl [Internet].

[13] Kornasiewicz O, Dudek K, Bugajski M, Najnigier B, Krawczyk M (2008). Choice of transplantation techniques and indications for liver transplantation in polycystic liver disease in patients with no signs of end-stage liver disease. Transplant Proc [Internet].

[14] Lauterio A, De Carlis R, Di Sandro S, Buscemi V, Andorno E, De Carlis L (2019). Delayed kidney transplantation in combined liver–kidney transplantation for polycystic liver and kidney disease. Transpl Int [Internet].

[15] Vauthey J (2002). Body surface area and body weight predict total liver volume in Western adults. Liver Transplant [Internet].

[16] Irazabal MV, Torres VE (2018). Total kidney volume and autosomal dominant polycystic kidney disease: a long-standing relationship. Am J Nephrol [Internet].

[17] Brezeanu LN, Brezeanu RC, Diculescu M, Droc G (2020). Anaesthesia for liver transplantation: an update. J Crit Care Med [Internet].

[18] Droc G, Jipa L (2018) Anesthesia for liver transplantation. In: Organ donation and transplantation - current status and future challenges [Internet]. InTech

[19] Eurotransplant. Chapter 5 - ET liver allocation system (ELAS). Eurotransplant Man [Internet]. 2015

[20] Jochmans I, Monbaliu D, Ceulemans LJ, Pirenne J, Fronek J (2017). Simultaneous liver kidney transplantation and (bilateral) nephrectomy through a midline is feasible and safe in polycystic disease. PLoS One [Internet].

[21] Lee TC, Cortez AR, Kassam A, Morris MC, Winer LK, Silski LS (2020). Outcomes of en bloc simultaneous liver-kidney transplantation compared to the traditional technique. Am J Transplant [Internet].

[22] Kälble T, Lucan M, Nicita G, Sells R, Revilla FJB, Wiesel M (2005). Eau guidelines on renal transplantation. Eur Urol.

[23] Shekhtman G, Huang E, Danovitch GM, Martin P, Bunnapradist S (2018). Combined dual-kidney liver transplantation in the United States: a review of United Network for Organ Sharing/Organ Procurement and Transplantation Network data between 2002 and 2012. Liver Transpl [Internet].

[24] Deutsche Stiftung Organtransplantation. Jahresbericht-organspende und transplantation in deutschland 2017 [Internet]. 2017 [cited 2019 Apr 9].

[25] Mehrabi A, Fonouni H, Ayoub E, Rahbari NN, Müller SA, Morath C, Seckinger J, Sadeghi M, Golriz M, Esmaeilzadeh M, Hillebrand N, Weitz J, Zeier M, Büchler MW, Schmidt J, Schmied BM (2009). A single center experience of combined liver kidney transplantation. Clin Transplant [Internet].

[26] Gunabushanam V, Clendenon J, Aldag E, Chadha M, Kramer D, Steers J, Sahajpal A (2016). En bloc liver kidney transplantation using donor splenic artery as inflow to the kidney: report of two cases. Am J Transplant.

[27] Nguyen MC, Black S, Washburn K, El-Hinnawi A (2018). En bloc liver-kidney transplantation with renal artery variation using donor splenic artery and left gastric artery as inflow to the kidney: case report. Int J Surg Case Rep [Internet].

[28] Halemani K, Bhadrinath N (2017). Combined liver and kidney transplantation: our experience and review of literature. Indian J Anaesth [Internet].

